# Association of the dietary index for gut microbiota with sleep disorder among US adults: the mediation effect of dietary inflammation index

**DOI:** 10.3389/fnut.2025.1528677

**Published:** 2025-03-17

**Authors:** Yingying Li, Fang Pan, Xiaofei Shen

**Affiliations:** Department of Otorhinolaryngology, Children’s Hospital of Nanjing Medical University, Nanjing, Jiangsu, China

**Keywords:** DI-GM, sleep disorder, DII, NHANES, cross-sectional study

## Abstract

**Background:**

Previous studies have confirmed the relationship between gut microbiota and sleep disorders, characterized by the persistent inability to achieve adequate sleep, with dietary composition playing a key role in maintaining microbiota homeostasis. Our study aims to explore the relationship between the newly proposed Dietary Index for Gut Microbiota (DI-GM) and sleep disorders, as well as whether the Dietary Inflammatory Index (DII) mediates this relationship.

**Methods:**

This study is based on data from 30,406 participants in the National Health and Nutrition Examination Survey (NHANES) from 2005 to 2018, a cross-sectional survey that represents the U.S. adult population. We used multivariable logistic regression models to examine the relationship between DI-GM and sleep disorders. Subgroup interaction analyses were conducted to assess the stability of the results. Mediation analysis was employed to explore the effect of the Dietary Inflammatory Index (DII) on the relationship between DI-GM and sleep disorders.

**Results:**

The DI-GM score was significantly negatively correlated with sleep disorders. After adjusting for covariates, each unit increase in DI-GM was associated with a 5% reduction in the prevalence of sleep disorders (*p* < 0.001). Additionally, there was a trend toward a decrease in the prevalence of sleep disorders with increasing DI-GM (trend *p* < 0.05). Dose–response curve analysis revealed a linear relationship between DI-GM and sleep disorders, with higher DI-GM scores being associated with lower prevalence of sleep disorders. DII was positively correlated with sleep disorders (*p* < 0.001) and decreased as DI-GM increased (*β* = −0.37, *p* < 0.001). Mediation analysis showed that DII significantly mediated the relationship between DI-GM and sleep disorders, with a mediation proportion of 27.36% (*p* < 0.001).

**Conclusion:**

The results of this study indicate that the DI-GM score was significantly negatively correlated with sleep disorders. A higher DI-GM score is associated with a lower incidence of sleep disorders, while the DII significantly mediated the relationship between DI-GM and sleep disorders. Specifically, an increase in DII may attenuate the protective effect of DI-GM on sleep disorders.

## Introduction

The persistent inability to achieve adequate sleep is a hallmark of sleep disorders, often manifested by insomnia, sleep apnea syndrome, narcolepsy, and restless legs syndrome (1). According to statistics, 35% of adults in the United States are affected by varying degrees of sleep disorders, and the Centers for Disease Control and Prevention (CDC) considers sleep disorders to be a public health epidemic (2, 3). In adults, sleep disorders can lead to daytime sleepiness, mood disturbances, and a decline in memory and motivation, which subsequently affect quality of life and work efficiency. In contrast, sleep disorders in children directly impact cognitive and behavioral development, potentially resulting in more severe consequences (4, 5). Furthermore, sleep disorders are considered risk factors for various diseases and are closely associated with increased mortality from many chronic conditions, placing a significant burden on public healthcare and the economy (6, 7).

Targeted interventions aimed at modulating the gut microbiota have been shown to have therapeutic effects on sleep disorders (8–10). Additionally, growing evidence suggests that dietary patterns are key factors influencing gut microbiota composition, with specific foods or food groups capable of inducing significant changes in the gut microbiome (11). Recently, Kase et al. developed a new dietary index (DI-GM) based on the literature (12). This index is strongly correlated biomarkers of gut microbiome diversity, enabling precise identification of dietary patterns that support microbial diversity. In contrast to HEI-2015 and modified Mediterranean Diet Score (MDS), DI-GM focuses on broader gut microbiome attributes (e.g., production of short-chain fatty acids (SCFAs), microbial phyla changes, and specific bacterial species), providing a more comprehensive assessment of the diet-microbiome relationship. Furthermore, by emphasizing specific foods rather than food categories, DI-GM allows for more targeted dietary recommendations, while demonstrating similar effectiveness as previous indices in evaluating overall dietary health.

The Dietary Inflammation Index (DII) is designed to assess the potential inflammatory effect of dietary components (13). It has been validated in numerous studies as an effective measure of the inflammatory impact of dietary patterns (14). Sleep is strongly linked to inflammation, with diet playing a crucial role in determining systemic inflammation levels. A systematic review has shown that an anti-inflammatory diet (i.e., a lower DII score) is linked to better outcomes in at least one aspect of sleep, with sleep efficiency and wake after sleep onset being the most reported (3). Moreover, a lower DII score has been shown to benefit gut microbiome diversity, which in turn may enhance sleep quality (15).

Thus, we designed a cross-sectional study, incorporating data from the National Health and Nutrition Examination Survey (NHANES) to analyze the relationship between DI-GM and sleep disorders. We hypothesize that a healthy diet not only promotes gut microbiota diversity but also improves sleep quality. Additionally, this study will explore whether the Dietary Inflammatory Index (DII) mediates this relationship.

## Methods

### Study participants

The National Health and Nutrition Examination Survey (NHANES) is based on a complex multi-stage sampling weighting design to obtain a representative sample of the non-institutionalized USA civilian population and is designed to assess the health and nutritional status of the non-institutionalized USA population. The NHANES research project has been approved by the Institutional Review Board (IRB) of the National Center for Health Statistics (NCHS), which ensures that the study adheres to ethical standards for the protection of participants’ rights and privacy (Accessed April 23, 2024).^1^

**Figure 1:**
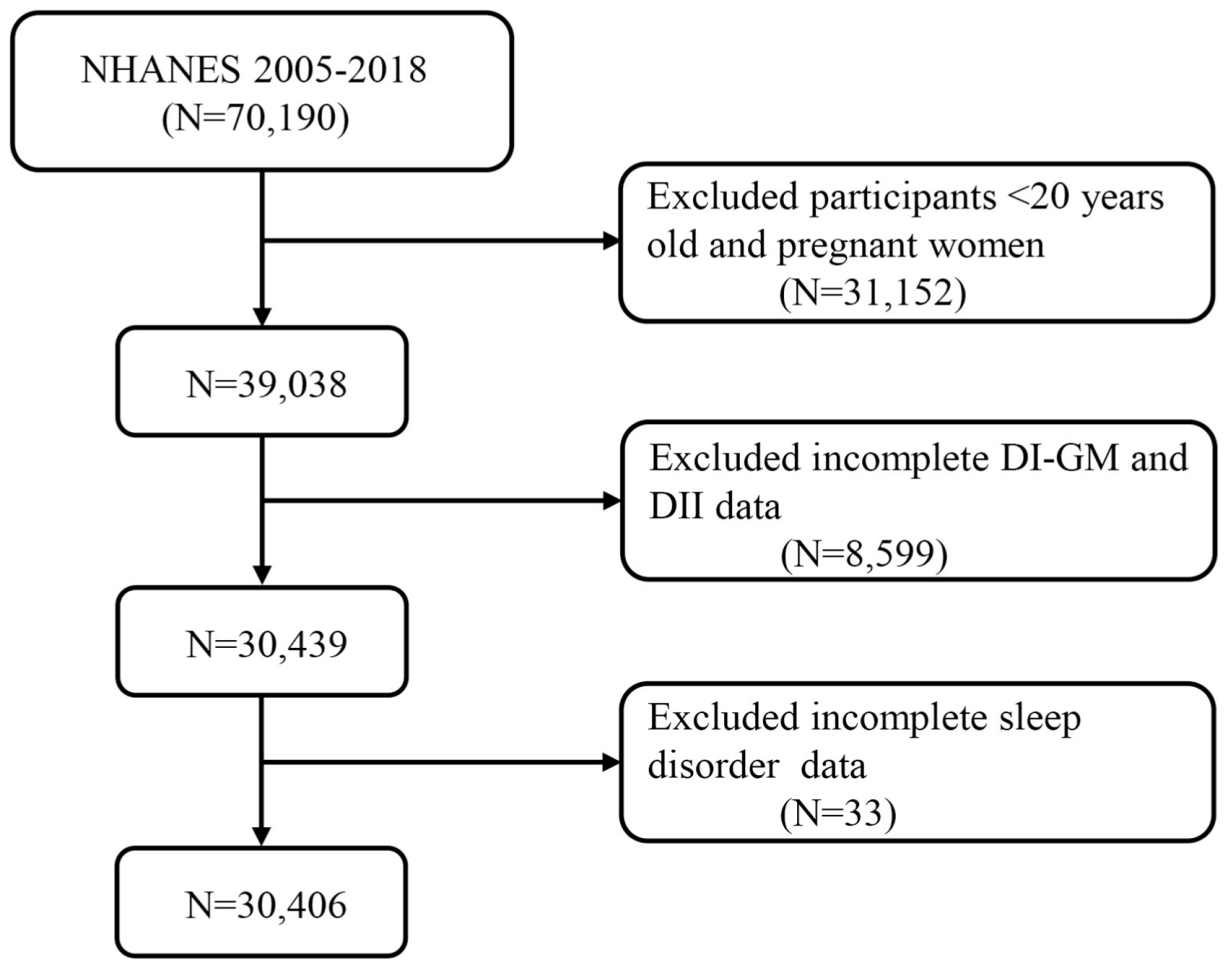
A flow diagram of eligible participant selection in the National Health and Nutrition Examination Survey. DI-GM, Dietary Index for Gut Microbiota; DII, Dietary Inflammatory Index.

We extracted data from a total of 70,190 participants in the NHANES dataset spanning from 2005 to 2018. After excluding individuals under 20 years of age, pregnant women (*n* = 31,152), as well as those with missing DI-GM data, DII data (*n* = 8,559), and incomplete questionnaire data on sleep disorders (*n* = 33), a final cohort of 30,406 participants was included (Figure 1).

### Characterization of DI-GM and DII

The DI-GM includes 14 types of foods and nutrients, with avocados, broccoli, chickpeas, coffee, cranberries, fermented dairy products, fiber, green tea, soy, and whole grains classified as beneficial components, while red meat, processed meat, refined grains, and high-fat diets (≥ 40% of energy from fat) are considered detrimental components (12). The DI-GM was calculated using dietary recall data from NHANES 2005–2018. For more detailed information regarding DI-GM, please refer to previous studies and Supplementary Table S1.

The Dietary Inflammation Index (DII) evaluates the inflammatory potential of an overall diet based on the pro-inflammatory or anti-inflammatory properties of individual dietary components, including vitamins and minerals (16). A higher DII score (≥ 0) indicates a pro-inflammatory diet, reflecting less healthy dietary habits, while a lower score (< 0) suggests an anti-inflammatory, healthier dietary pattern. The formula used to calculate the DII score is outlined in the Supplementary materials.

### Diagnosis of sleep disorder

According to previous studies (17, 18), the criteria for diagnosing sleep disorders were based on the response to the question “Has a doctor ever told you that you have a sleep disorder?” in the NHANES personal interview questionnaire. Individuals who answered “Yes” were defined as having a sleep disorder (19). For further details, please refer to the NHANES website.

### Covariables

We constructed a directed acyclic graph (DAG) (20) to visualize the hypothesized associations of the primary exposure (Dietary Index for Gut Microbiota) with the outcomes of interest (the prevalence of sleep disorder), and potential covariates. Age, sex, race, marital status, education level, poverty-to-income ratio (PIR), hypertension, diabetes, and hyperlipidemia-were included in the multivariable-adjusted models based on previous relevant research (21–23). Detailed information regarding these covariates can be found in Supplementary Table S2. The resulting DAG is presented in Supplementary Figure S1.

### Statistical analysis

Data were analyzed statistically using R (version 4.3.1). During the analysis, we applied the weights recommended by the National Center for Health Statistics (NCHS) to ensure that the data from our survey were nationally representative. Both types of data in this study were analyzed using a weighted calculation method. The weighting variable was the two-day dietary sample weight (WTDR 2D), and the weight calculation formula for 2005–2018 was 1/7 × WTDR 2D. Continuous variables are presented as means ± standard deviations, with *p*-values determined by the weighted Student’s t-test. For categorical variables (weighted *N*, %), *p*-values were computed using the weighted chi-square test (24). The relationship between DI-GM and sleep disorders was assessed using multiple multivariable logistic regression models. Additionally, DI-GM was divided into three categories. To ensure the consistency of the relationship, a trend test was performed, and *p*-values were calculated.

During the analysis, we constructed three multivariable logistic regression models, ranging from an unadjusted model to progressively adjusted models that accounted for confounding factors, including demographic variables and chronic diseases, to comprehensively evaluate the association between DI-GM and sleep disorders. Model 1 was the unadjusted model, Model 2 further adjusted for age, gender, race, education level, and the poverty-to-income ratio (PIR), and Model 3 was the fully adjusted model, which additionally controlled for hypertension, diabetes, and hyperlipidemia. We further explored potential nonlinear relationships through smooth curve fitting and conducted mediation analysis using bootstrap resampling to quantify the mediating effect of DII on the association.

Additionally, the odds ratios (ORs) corresponding to each standard deviation increase in DI-GM were calculated. In the subgroup analyses, we also adjusted for age, sex, race, marital status, education level, PIR, hypertension, diabetes, and hyperlipidemia to further validate the robustness of our findings.

**Figure 2:**
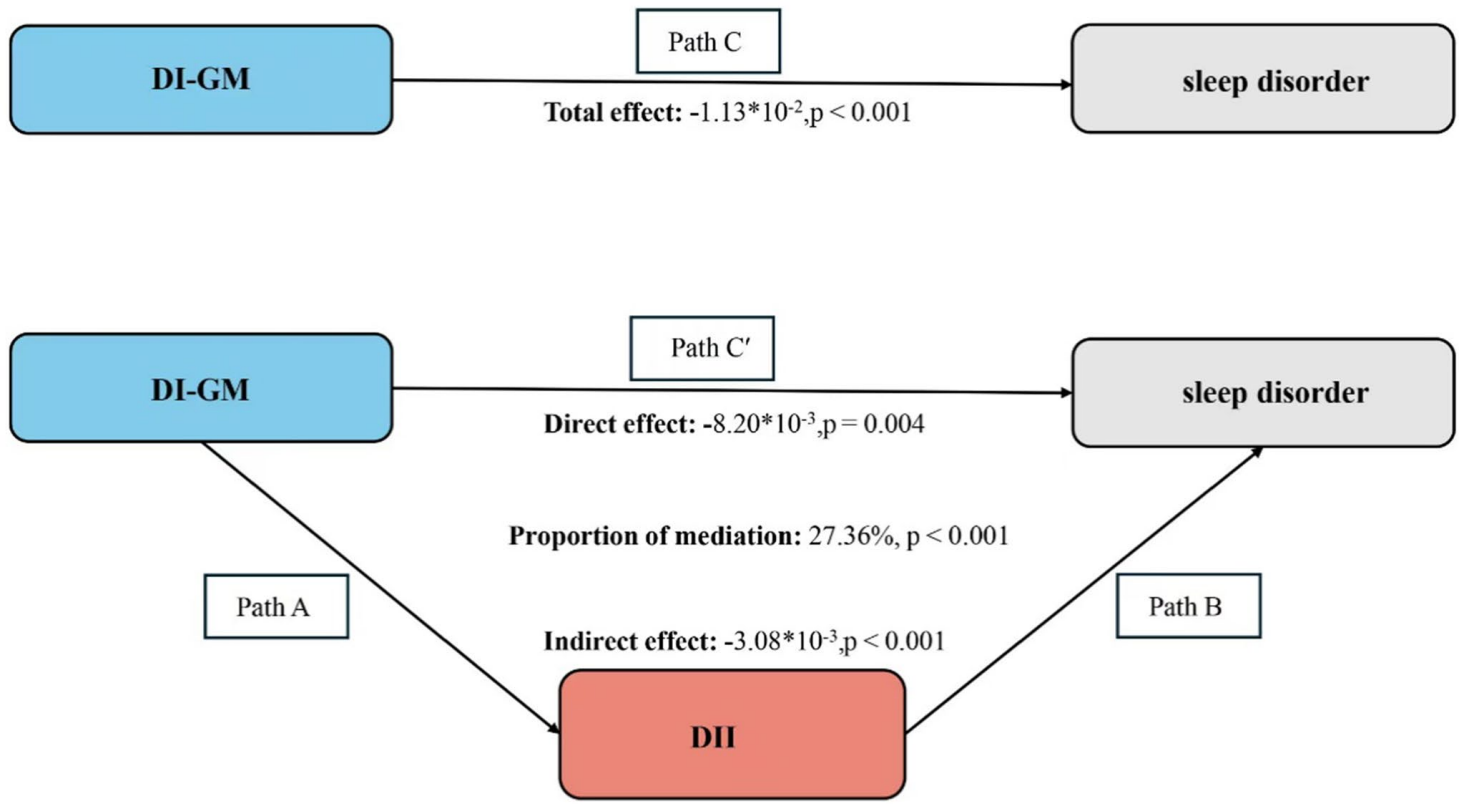
Schematic diagram of the mediation effect analysis. Path C indicates the total effect; path C′ indicates the direct effect. The indirect effect is estimated as the multiplication of paths A and B (path A*B). The mediated proportion is calculated as indirect effect/ (indirect effect + direct effect) × 100%. DI-GM, Dietary Index for Gut Microbiota; DII, Dietary Inflammatory Index. Analyses were adjusted for age, sex, education level, marital, PIR, race, hypertension, diabetes, and hyperlipidemia.

Finally, the ‘mediation’ package in R software was used to calculate the indirect, direct, and total effects of DI-GM, DII, and sleep disorders. Mediation analysis was conducted using 1,000 bootstrap resamples and variable adjustments to determine whether DII mediates the relationship between DI-GM and sleep disorders. The formula for calculating the mediation effect is: Indirect effect/ (Indirect effect + Direct effect) × 100% (25). The total effect of DI-GM on sleep disorders (path C), the direct effect of DI-GM on sleep disorders including DII as a mediator (path C′), the effect of DI-GM on DII (path A), the effect of DII on sleep disorders (path B), and the indirect effect of DII on the relationship between DI-GM and sleep disorders (path A*B) are all represented by regression coefficients (Figure 2).

## Results

### Baseline characteristics

This study included 30,406 participants, representing 135,090,633 US residents. The prevalence of sleep disorders was 17% (equivalent to 22,322,314 individuals). Compared to other racial groups, the prevalence of sleep disorders was higher among “Non-Hispanic White people (67%). Participants with a high school education or higher had a higher prevalence of sleep disorders (84%). The prevalence of sleep disorders was higher among individuals with hypertension, diabetes, or hyperlipidemia (*p* < 0.05). Additionally, compared to the non-sleep disorder group, participants in the sleep disorder group had lower DI-GM and higher DII scores (*p* < 0.05). Baseline characteristics are detailed in Table 1.

**Table 1:** Baseline characteristics of all participants were stratified by sleep disorder, weighted.

Characteristic	Overall, *N* = 135,090,633 (100%)	Non-sleep disorder, *N* = 112,768,319 (83%)	Sleep disorder, *N* = 22,322,314 (17%)	*p*-value
No. of participants in the sample	30,406	25,848	4,558	**-**
Age (%)				**<0.001**
20–40	49,626,663 (37%)	44,136,357 (39%)	5,490,305 (25%)	
41–60	49,086,295 (36%)	39,559,425 (35%)	9,526,870 (43%)	
> 60	36,377,675 (27%)	29,072,536 (26%)	7,305,139 (33%)	
Gender (%)				**0.030**
Female	69,193,358 (51%)	57,259,909 (51%)	11,933,449 (53%)	
Male	65,897,274 (49%)	55,508,409 (49%)	10,388,865 (47%)	
Race (%)				**<0.001**
Non-Hispanic White	90,718,086 (67%)	74,647,273 (66%)	16,070,813 (72%)	
Other	17,964,530 (13%)	15,238,473 (14%)	2,726,057 (12%)	
Non-Hispanic Black	15,310,331 (11%)	12,968,322 (11%)	2,342,009 (10%)	
Mexican American	11,097,685 (9%)	9,914,250 (9%)	1,183,434 (6%)	
Married/live with partner (%)				0.228
No	50,932,517 (38%)	42,264,503 (37%)	8,668,015 (39%)	
Yes	84,119,754 (62%)	70,468,861 (63%)	13,650,892 (61%)	
Education level (%)				**<0.001**
Below high school	21,139,910 (16%)	18,381,697 (16%)	2,758,213 (12%)	
High School or above	113,869,472 (84%)	94,308,631 (84%)	19,560,841 (88%)	
PIR (%)				0.998
Poor	27,589,076 (22%)	22,999,492 (22%)	4,589,584 (22%)	
Not Poor	97,567,568 (78%)	81,338,881 (78%)	16,228,687 (78%)	
Hypertension (%)				**<0.001**
No	82,167,748 (61%)	72,026,344 (64%)	10,141,404 (45%)	
Yes	52,920,617 (39%)	40,739,707 (36%)	12,180,910 (55%)	
Diabetes (%)				**<0.001**
No	115,058,040 (85%)	97,972,935 (87%)	17,085,105 (77%)	
Yes	20,032,592 (15%)	14,795,383 (13%)	5,237,209 (23%)	
Hyperlipidemia (%)				**<0.001**
No	42,019,307 (31%)	36,638,934 (32%)	5,380,373 (24%)	
Yes	93,068,524 (69%)	76,127,890 (68%)	16,940,634 (76%)	
DI-GM (mean (SD))	5.03 (1.72)	5.05 (1.73)	4.95 (1.67)	**0.013**
DI-GM, Tertile (%)				0.109
T1	24,689,539 (18%)	20,420,600 (18%)	4,268,939 (19%)	
T2	59,139,401 (44%)	49,067,676 (44%)	10,071,725 (45%)	
T3	51,261,693 (38%)	43,280,043 (38%)	7,981,650 (36%)	
DII (mean (SD))	1.30 (1.87)	1.27 (1.87)	1.46 (1.85)	**<0.001**
DII, Tertile (%)				**0.002**
T1	45,030,564 (33%)	38,246,304 (34%)	6,784,261 (30%)	
T2	45,031,401 (33%)	37,684,545 (33%)	7,346,856 (33%)	
T3	45,028,667 (33%)	36,837,470 (33%)	8,191,197 (37%)	

Mean (SD) for continuous variables: the *p* value was calculated by the weighted Students T-test. Percentages (weighted *N*, %) for categorical variables: the *p* value was calculated by the weighted chi-square test. DI-GM, Dietary Index for Gut Microbiota; DII, Dietary Inflammatory Index; PIR, poverty income ratio. Bold values indicate statistically significant results (*p* < 0.05).

### Relationship between DI-GM and sleep disorder

**Figure 3:**
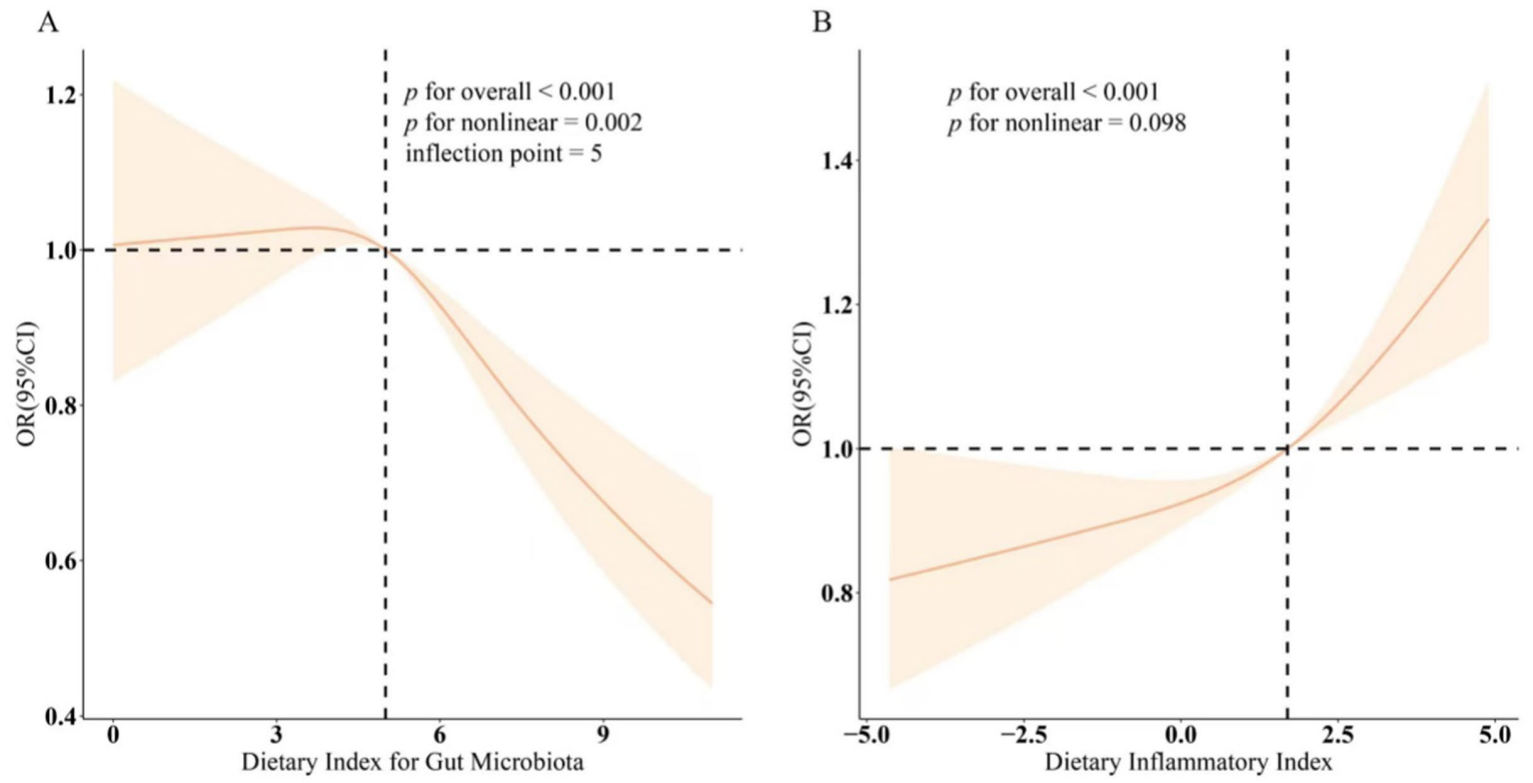
Dose–response relationships between DI-GM, DII, and sleep disorder. **(A)** DI-GM – sleep disorder. **(B)** DII – sleep disorder. OR (solid lines) and 95% confidence levels (shaded areas) were adjusted for age, sex, education level, marital, PIR, race, hypertension, diabetes, and hyperlipidemia.

Table 2 presents the results of a multivariable logistic regression analysis, which showed a significant association between DI-GM, DII, and sleep disorders (*p* < 0.001). In the fully adjusted model, we found a negative association between DI-GM and the prevalence of sleep disorders (OR 0.95, 95% CI 0.92–0.98). This suggests that for each unit increase in DI-GM, there is a 5% reduction in the prevalence of sleep disorders. The authors further transformed DI-GM from a continuous variable into a categorical variable, dividing it into three tertiles. The average prevalence of sleep disorders in the highest tertile group of DI-GM was 0.18 units lower than in the lowest tertile group (OR 0.82, 95% CI 0.71–0.93), and the trend test within the model confirmed this finding (*p* < 0.05). Additionally, Model 3 revealed a positive association between DII and sleep disorders, with the most pronounced difference in the highest tertile group: T3 (OR 1.25, 95% CI 1.10–1.42). Finally, the smoothing curve fitting results demonstrated a nonlinear association between DI-GM, DII, and sleep disorders, suggesting a negative nonlinear correlation between DI-GM and sleep disorders (nonlinearity = 0.002) and a positive nonlinear correlation between DII and sleep disorders (nonlinearity = 0.098), as shown in Figure 3.

**Table 2:** Association between DI-GM, DII, and sleep disorder, NHANES 2005–2018.

Characteristics	Model 1 [OR (95% CI)]	*p*-value	Model 2 [OR (95% CI)]	*p*-value	Model 3 [OR (95% CI)]	*p*-value
DI-GM – sleep disorder						
Continuous	0.97 (0.94,0.99)	0.010	0.93 (0.91,0.96)	<0.001	0.95 (0.92,0.98)	<0.001
Tertile						
T1	1 (ref.)		1 (ref.)		1 (ref.)	
T2	0.98 (0.86,1.12)	0.780	0.92 (0.80,1.06)	0.260	0.95 (0.82,1.08)	0.410
T3	0.88 (0.77,1.01)	0.060	0.76 (0.67,0.88)	<0.001	0.82 (0.71,0.93)	0.004
*P* for trend	0.040		<0.001		0.002	
DII – sleep disorder						
Continuous	1.06 (1.03,1.09)	<0.001	1.07 (1.04,1.10)	<0.001	1.06 (1.03,1.09)	<0.001
Tertile						
T1	1 (ref.)		1 (ref.)		1 (ref.)	
T2	1.10 (0.96,1.26)	0.160	1.13 (0.98,1.30)	0.090	1.10 (0.95,1.27)	0.190
T3	1.25 (1.10,1.42)	<0.001	1.32 (1.16,1.50)	<0.001	1.25 (1.10,1.42)	<0.001
*P* for trend	<0.001		<0.001		<0.001	

Model 1: no covariates were adjusted. Model 2: age, sex, education level, marital, PIR, and race were adjusted. Model 3: age, sex, education level, marital, PIR, race, hypertension, diabetes, and hyperlipidemia were adjusted. DI-GM, Dietary Index for Gut Microbiota; DII, Dietary Inflammatory Index; PIR, poverty income ratio; OR, odds ratio; CI, confidence interval.

### Subgroup analysis

**Figure 4:**
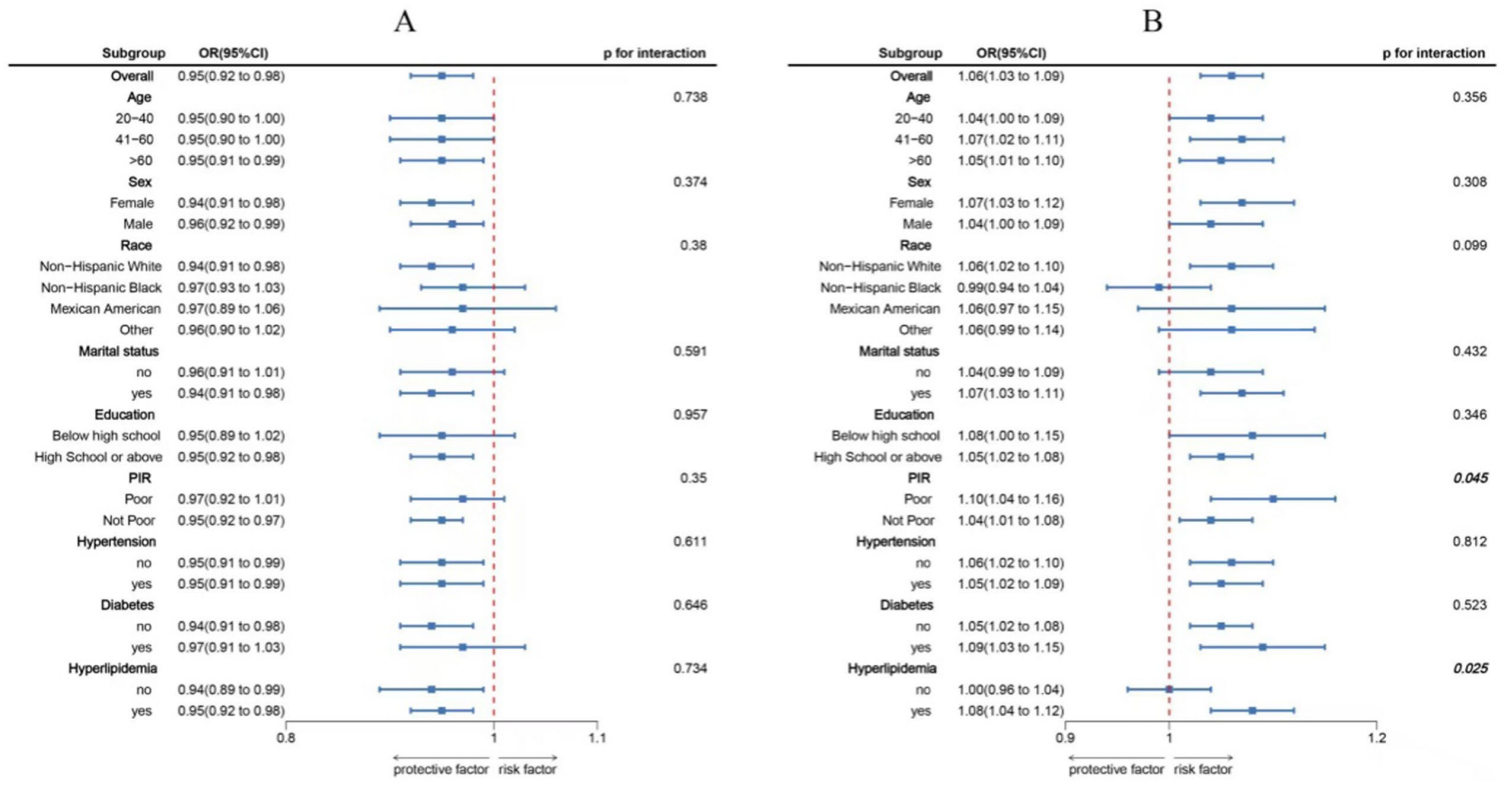
Subgroup analysis between DI-GM, DII, and sleep disorder. **(A)** DI-GM-sleep disorder. **(B)** DII-sleep disorder. ORs were calculated as each standard deviation increased in DI-GM/DII. Analyses were adjusted for age, sex, education level, marital, PIR, race, hypertension, diabetes, and hyperlipidemia.

Figure 4 illustrates the subgroup analysis of how DI-GM is associated with sleep disorders, adjusting for and stratifying by age, gender, education level, marital status, income, race, hypertension, diabetes, and hyperlipidemia. This analysis further explores the stability of the association between DI-GM, DII, and sleep disorders, as well as potential interactions. In the subgroup analysis, no significant interactions were found between DI-GM and these stratifying variables (*p* > 0.05), and the negative association remained stable. However, in the analysis of the association between DII and sleep disorders, significant interactions were observed with PIR and hyperlipidemia.

### Mediation effect analysis

In Model 3, after adjusting for all covariates, the prevalence of sleep disorders in the highest tertile (T3) of DII was 25% higher compared to the first tertile (T1) (OR 1.25, 95% CI 1.10–1.42). Furthermore, when DII was treated as a continuous variable, its positive association with sleep disorders remained statistically significant (OR 1.06, 95% CI 1.03–1.09), as shown in Table 1. After adjusting for all covariates, there was a significant statistical correlation between DI-GM and DII (*β* = −0.37, 95% CI: −0.38 to −0.35, *p* < 0.001) (Table 3). Based on the assumptions of the mediation analysis; after adjusting for all covariates, we observed a mediating effect of DII (Figure 2). The indirect effect of DII (indirect effect = −3.08 × 10^−3^, *p* < 0.001; direct effect = −8.2 × 10^−3^, *p* = 0.004) mediated 27.36% of the association between DI-GM and sleep disorders (mediating proportion = indirect effect/ (indirect effect + direct effect) × 100%, *p* < 0.001).

**Table 3:** Multivariate linear regression of DI-GM and DII.

	*β*	95%CI	*p*-value
DI-GM – DII	−0.37	(−0.38, −0.35)	<0.001

Adjusted for age, sex, education level, marital, PIR, race, hypertension, diabetes, and hyperlipidemia.

## Discussion

This large population-based study found a significant negative association between DI-GM and the prevalence of sleep disorders, independent of a range of confounding factors such as gender, age and ethnicity. Furthermore, the DII seems to serve as an intermediary factor in the association between DI-GM and sleep disorders. These findings suggest that a higher DI-GM is associated with a lower prevalence of sleep disorders, with DII mediating this relationship, supporting our hypothesis. Results from smooth curve fitting and subgroup analyses further confirmed the stability of these findings.

Sleep is an essential and complex physiological process that plays a critical role in maintaining both our physical and mental health (26). However, sleep disorders affect nearly 20% of adults worldwide, contributing to various chronic cardiovascular and endocrine diseases (27, 28), and are now recognized as a global public health issue (29). Research suggests that the microbiota-gut-brain (MGB) axis can directly or indirectly modulate sleep behavior. For instance, one study found that depletion of the gut microbiota in mice led to disruption of their sleep architecture, as changes in the gut microbiota affected intestinal neurotransmitters, thereby influencing the sleep/wake rhythm of the mice (30). Other studies have also confirmed that metabolites produced by the gut microbiota, such as *γ*-aminobutyric acid (GABA), dopamine, and serotonin (5-HT), can directly influence sleep rhythms (31, 32). A randomized controlled trial (RCT) demonstrated that supplementation with probiotics significantly improved sleep quality (33).

Furthermore, a recent study published in Cell Press confirmed that the gut microbiota can also regulate sleep through another pathway: the endocrine route, specifically the hypothalamic–pituitary–adrenal (HPA) axis. Impairment of gut microbiota stability interferes with stress response pathways in the hippocampus and amygdala, leading to dysregulation of the circadian pacemaker in the brain, disturbances in glucocorticoid rhythms, and excessive activation of the HPA axis during sleep/wake transitions, which ultimately results in arousal and insomnia, which is the most common manifestation of sleep disorders (34, 35). The influence of the gut microbiota on the HPA axis can persist throughout the human lifespan, while the HPA axis, in turn, can regulate the MGB axis through cortisol secretion (36). Therefore, the homeostasis of the gut microbiota exerts a profound impact on sleep. Some of our research findings support the above conclusions from a population-based perspective, demonstrating that foods beneficial to gut microbiota diversity such as avocados, broccoli, chickpeas, coffee, cranberries, fermented dairy, fiber, green tea, soybeans, and whole grains, are associated with fewer sleep problems. Additionally, we focused on specific foods rather than broad food categories, which makes the research findings more easily translatable to clinical and public health decision-making.

Dietary modifications and probiotic supplementation are two major interventions for regulating the gut microbiota, with dietary adjustments being the core component. Gut microbiota utilizes dietary fiber as a fermentation substrate to produce beneficial short-chain fatty acids (SCFAs), thereby exerting their effects (37). A reduction in dietary fiber intake has been shown to decrease the richness and diversity of the gut microbiota (38, 39). Animal experiments by H. Shi et al. revealed that mice deprived of dietary fiber for 15 weeks exhibited significant gut dysbiosis (reduced Bacteroidetes and increased Firmicutes), along with impaired neurocognitive function (40). In contrast, consuming more fiber boosts the population of beneficial bacteria such as Prevotella and Bifidobacterium, thereby promoting brain health (41, 42). Additionally, a healthy dietary structure is also beneficial for maintaining gut microbiota balance. The Mediterranean diet (MD), defined by a high consumption of fresh fruits, vegetables, legumes, and whole grains, has been extensively studied and shown to improve metabolic function and reduce chronic inflammation through its favorable effects on the gut microbiota (43, 44). Studies have confirmed that MD increases the abundance of Bacteroides, Bifidobacterium, and Prevotella (45, 46). Conversely, high intake of calories, fats, and processed proteins reduces beneficial Bifidobacterium and increases the abundance of Firmicutes, which are pro-inflammatory (47). A randomized controlled trial by S. Hoscheidt et al. demonstrated that after 4 weeks of adherence to MD, cognitively normal individuals showed improvements in brain perfusion and neurocognitive function (48). Furthermore, a recent meta-analysis confirmed that higher adherence to MD is associated with better sleep features (49). This aligns with our study’s findings, where we observed a lower prevalence of sleep disorders in individuals who preferred whole grains, legumes, fiber, and fresh vegetables, while consuming fewer processed meats and refined grains. This is likely attributable to DI-GM including fiber, whole grains, legumes, and fresh vegetables, all of which are food categories advocated by traditional MD.

The DI-GM framework used in this study also includes probiotic supplementation (fermented dairy products) as part of the scoring criteria. Probiotic supplementation consists of live microorganisms that confer health benefits to the host upon adequate consumption, with their origin typically traced to food production or storage methods (50). Clinical studies have confirmed the positive outcomes of Bifidobacterium and Lactobacillus on neural function (51). Research has shown that daily supplementation with *Lactobacillus acidophilus* for 5 weeks can improve sleep quality (52). Animal experiments by Y. Wang et al. also demonstrated that *Lactobacillus plantarum* MA2 could modulate the TLR4/MYD88/NLRP3 signaling pathway to prevent neuroinflammation (53). Another study found that oral supplementation with *Lactobacillus reuteri* for 9 weeks effectively increased vitamin D levels in circulation, thereby improving sleep (54). A recent meta-analysis further confirmed previous findings, showing that probiotics have a positive effect on individuals with sleep disorders or poor sleep quality (55). This study, by employing the latest DI-GM tool, explores the combined effects of diet and microbiota on sleep disorders, providing a more comprehensive understanding of how dietary patterns and gut microbiota balance influence neurological functions, including sleep and cognition.

DII is widely used to assess the inflammatory effects of dietary patterns (56). A recent cohort study involving 17,637 European adults confirmed that an increase in the DII is associated with elevated expression of inflammatory markers such as C-reactive protein (CRP), interleukins (IL-6), and tumor necrosis factor-*α* (TNF-α) (57). Similarly, a cross-sectional study conducted on European adolescents revealed a strong relationship between the DII score and the expression of inflammatory markers such as TNF-α, IL-1, and IL-2 (58). Sleep is regulated by various inflammatory factors (59), and studies have found that the expression of IL-1β is positively correlated with the duration of non-REM (NREM) sleep (60). Elevated levels of TNF-α are known to enhance the depth of physiological sleep. The NF-κB signaling pathway plays a key role in mediating this effect, which in turn increases the expression of nitric oxide synthase (NOS), cyclooxygenase-2 (COX-2), and adenosine A1 receptors. These molecules play key roles in regulating sleep by influencing neuronal activity and promoting sleep induction and maintenance. Specifically, nitric oxide and COX-2 are involved in sleep regulation by affecting the central nervous system, while adenosine A1 receptors contribute to sleep promotion, particularly during the NREM sleep phase (61). In addition, a higher Dietary Inflammatory Index (DII) can lead to an increase in pro-inflammatory microbes in the gut, which subsequently promotes the release of inflammatory factors. This cascade of inflammation can disrupt sleep architecture (15, 62). Our findings are consistent with previous studies.

Our subgroup analysis revealed an interaction between PIR and hyperlipidemia in the relationship between DII and sleep disorders, while no such interaction was found between covariates and the relationship between DI-GM and sleep disorders. These results highlight the potential benefits of increasing the dietary intake of foods that nourish the gut microbiota in improving sleep in the general population, with a particular focus on health education for low-income groups and individuals with hyperlipidemia. Additionally, our study demonstrated that the DII mediated the inverse relationship between DI-GM and the incidence of sleep disorders, suggesting that a diet with reduced inflammation may contribute to improved sleep (63).

This study provides the first evidence of a link between the DI-GM and sleep disorders. The findings highlight the importance of a health-promoting dietary pattern—such as increasing the intake of broccoli, coffee, fermented dairy, fiber, green tea, and whole grains—that benefits gut microbiota. These results offer a theoretical foundation for incorporating gut microbiota factors into dietary guidelines. Based on this, we propose dietary interventions as a potential strategy to alleviate sleep disorders. The study is based on the NHANES dataset, which provides nationally representative data and accounts for sample weights, making the conclusions reliable and highly generalizable. We also adjusted for confounding factors and covariates, conducting subgroup analyses to examine the stability of the connection between DI-GM and sleep disorders across diverse population groups. Finally, we also identified the mediating role of the Dietary Inflammatory Index (DII) in the relationship between DI-GM and sleep disorders for the first time (64). However, our study has certain limitations. Initially, the cross-sectional design of this study limits our ability to draw causal conclusions between DI-GM and sleep disorders. To confirm our results, future longitudinal research is necessary. Second, despite adjusting for multiple confounders, it is not possible to eliminate the influence of all potential confounding factors. Moreover, DI-GM may not encompass all beneficial foods related to gut microbiota, and the NHANES dietary and sleep questionnaire data are self-reported, which may introduce recall bias. Lastly, our findings are based on the US population, and further studies in diverse populations are needed to validate and expand our results.

## Conclusion

This study identified a significant negative correlation between the DI-GM, a novel index that effectively characterizes dietary patterns favorable to gut microbiota diversity, and sleep disorders. Furthermore, mediation analysis revealed the mediating role of the Dietary Inflammatory Index (DII) in this relationship. The findings further underscore the role of maintaining a healthy diet and gut microbiota homeostasis in improving sleep quality, highlighting the importance of a balanced dietary structure for gut microbiota diversity and sleep health. However, these conclusions require validation through large-scale prospective studies. In summary, studying dietary health, gut microbiota homeostasis, and sleep quality as an integrated whole could benefit a broader population and reduce the global disease burden.

## Data Availability

Publicly available datasets were analyzed in this study. This data can be found at: https://wwwn.cdc.gov/nchs/nhanes/.

## Footnotes

^1^ https://www.cdc.gov/nchs/nhanes/index.htm
