# Supplementary material for: Association of the dietary index for gut microbiota with sleep disorder among US adults: the mediation effect of dietary inflammation index
Source: Front Nutr. 2025 Mar 17;12:1528677. doi: 10.3389/fnut.2025.1528677 (PMC11955485; doi:10.3389/fnut.2025.1528677)
Supplement: Supplementary file 1 [file Data_Sheet_1.docx]

**Supplementary Material**

**Supplementary Table 1. Definition of components in DI-GM.**

| **Component** | **Included Foods within the Component** | **Scoring** |
| --- | --- | --- |
| **Beneficial to gut microbiota** |  |  |
| Avocados | Avocados | For each component, a score of 1 if consumption at or above the sex-specific median, else 0 |
| Broccoli | Broccoli |  |
| Chickpea | Chickpeas |  |
| Coffee | Coffee |  |
| Cranberries | Cranberries |  |
| Fermented dairy | Yogurt, cheese, kefir, sour cream, buttermilk |  |
| Fiber | Not applicable |  |
| Green Tea | Green Tea |  |
| Soybean | Soy products—Soy milk, Tofu |  |
| Whole grains | Grains defined as whole grains, containing the entire grain kernel―the bran, germ, and endosperm |  |
| **Unfavorable to gut microbiota** |  |  |
| High-fat diet (% energy) | Not applicable | 0 if consumption at or above 40% energy from fat, else 1 For each remaining component, a score of 0 if consumption at or above the sex-specific median, else 1 |
| Processed meat | Frankfurters, sausages, corned beef, and luncheon meat that are made from beef, pork, or poultry |  |
| Red meat | Beef, veal, pork, lamb, and game meat; excludes organ meat and cured meat |  |
| Refined grains | Refined grains that do not contain all of the components of the entire grain kernel |  |

DI-GM Scoring Method: Using dietary data from the NHANES database, the sex-specific median intake for each component listed in the table above was first calculated. Then, a score of 1 was assigned to participants whose consumption of each beneficial component exceeded the sex-specific median, and to those whose consumption of each unfavorable component was below the sex-specific median. A score of 0 was assigned to participants whose consumption of each beneficial component was below the sex-specific median, and to those whose consumption of each unfavorable component exceeded the sex-specific median. The scores for each component were summed to obtain a DI-GM score ranging from 0 to 14[1].

**Supplementary Table 2. Description of covariates**

| Covariates | Description in NHANES |
| --- | --- |
| Age | Divided into three groups: 20-40 years old, 41-60  years old, >60 years old |
| Gender | Male and Female |
| Race | Mexican American, Non-Hispanic Black, Non-Hispanic White, Other Race |
| Educational level | Below high school, High School or above |
| Marital status | Yes: Married/Living with partner |
| PIR | Poor: <1.3; Not Poor:>=1.3 |
| Hyperlipidemia | (1) Triglyceride (TG) levels ≥150 mg/dl (1.7 mmol/L);(2) Total cholesterol (TC) levels ≥200 mg/dl (5.18 mmol/L);(3) Low-density lipoprotein (LDL) levels ≥130 mg/dl (3.37 mmol/L);(4) High-density lipoprotein (HDL) levels: Men: <40 mg/dl (1.04 mmol/L); Women: <50 mg/dl (1.30 mmol/L) ;(5) Individuals taking cholesterol-lowering drugs are also considered hyperlipidemia. |
| Diabetes | Diabetes was defined as a history of previous diabetes, HbA1c level ≥6.5%, or fasting blood glucose level ≥126 mg/dL |
| Hypertension | The diagnostic criteria consist of self-reported hypertension history, the utilization of antihypertensive medication, a systolic blood pressure (SBP) ≥ 140mmHg, or a diastolic blood pressure (DBP) ≥ 90mmHg |

PIR, Ratio of family income to poverty.

**Calculation of the dietary inflammatory index[2]**.

Calculation of the DII is based on dietary intake data that are then linked to the regionally representative world database that provided a robust estimate of a mean and standard deviation for each parameter. These then become the multipliers to express an individual's exposure relative to the ‘standard global mean’ as a Z-score. This is achieved by subtracting the ‘standard mean’ from the amount reported and dividing this value by its standard deviation. To minimize the effect of ‘right skewing’, this value is converted to a percentile score. To achieve a symmetrical distribution centered around 0 (null), with values bounded between −1 (maximally anti-inflammatory) and +1 (maximally pro-inflammatory), each percentile score is doubled, and then 1 is subtracted.

The centered percentile value for each food parameter is then multiplied by its respective ‘overall food parameter-specific inflammatory effect score’ to obtain the ‘food parameter-specific DII score’. Finally, all the ‘food parameter-specific DII scores’ are summed to create the ‘overall DII score’ for an individual.

1. Kase, B.E., et al., *The Development and Evaluation of a Literature-Based Dietary Index for Gut Microbiota.* Nutrients, 2024. **16**(7).

2. Shivappa, N., et al., *Designing and developing a literature-derived, population-based dietary inflammatory index.* Public Health Nutr, 2014. **17**(8): p. 1689-96.


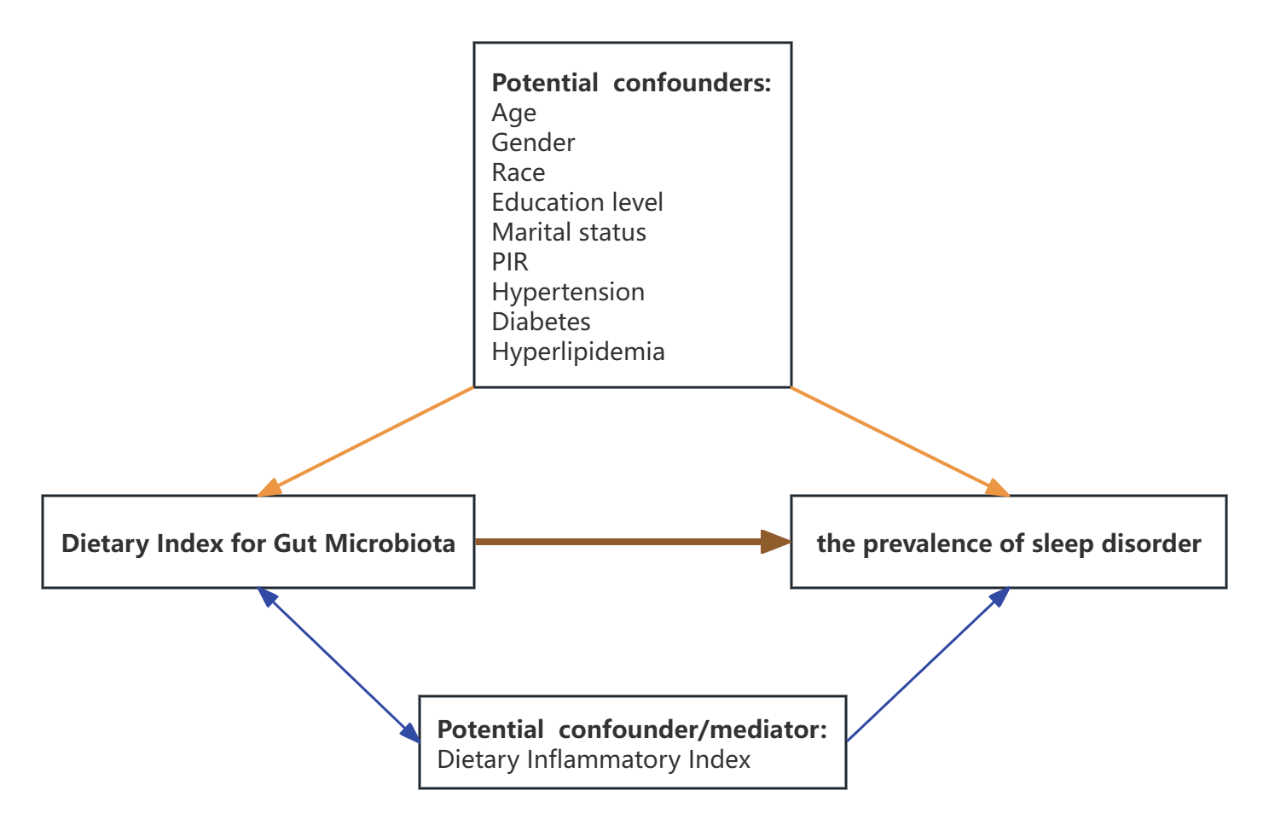


**Figure S1.** The hypothetical directed acyclic graph used to select potential covariates.
